# Rapid-SL identifies synthetic lethal sets with an arbitrary cardinality

**DOI:** 10.1038/s41598-022-18177-w

**Published:** 2022-08-18

**Authors:** Mehdi Dehghan Manshadi, Payam Setoodeh, Habil Zare

**Affiliations:** 1 Department of Chemical Engineering, School of Chemical, Petroleum and Gas Engineering, Shiraz University, Shiraz, Iran; 2 Glenn Biggs Institute for Alzheimer’s & Neurodegenerative Diseases, 7400 Merton Minter, San Antonio, TX 78229 USA; 3 Department of Cell Systems and Anatomy, University of Texas Health Science Center, San Antonio, San Antonio, TX USA

**Keywords:** Computer modelling, Target identification, Systems biology, Biochemical reaction networks

## Abstract

The multidrug resistance of numerous pathogenic microorganisms is a serious challenge that raises global healthcare concerns. Multi-target medications and combinatorial therapeutics are much more effective than single-target drugs due to their synergistic impact on the systematic activities of microorganisms. Designing efficient combinatorial therapeutics can benefit from identification of synthetic lethals (SLs). An SL is a set of non-essential targets (i.e., reactions or genes) that prevent the proliferation of a microorganism when they are “knocked out” simultaneously. To facilitate the identification of SLs, we introduce Rapid-SL, a new multimodal implementation of the Fast-SL method, using the depth-first search algorithm. The advantages of Rapid-SL over Fast-SL include: (a) the enumeration of all SLs that have an arbitrary cardinality, (b) a shorter runtime due to search space reduction, (c) embarrassingly parallel computations, and (d) the targeted identification of SLs. Targeted identification is important because the enumeration of higher order SLs demands the examination of too many reaction sets. Accordingly, we present specific applications of Rapid-SL for the efficient targeted identification of SLs. In particular, we found up to 67% of all quadruple SLs by investigating about 1% of the search space. Furthermore, 307 sextuples, 476 septuples, and over 9000 octuples are found for *Escherichia coli* genome-scale model, iAF1260*.*

## Introduction

A number of human pathogenic microorganisms show multidrug resistance, which is a serious challenge in the era of global healthcare^1,2^. Most of these species benefit from several pathogenicity factors (i.e., the production of antigens) and broad drug-resistance mechanisms (i.e., antibiotic target mutations). Hence, disrupting the activity of only a single gene in these microorganisms does not guarantee to prevent their growth or the biosynthesis of virulence factors. Furthermore, targeting the essential reactions or genes in some pathogens may cause a significant increase in biofilm-associated reactions. This implies that single essential genes may not be proper targets for these types of microorganisms^3^. In contrast, multi-target medications and combinatorial therapeutics synergistically impress the microorganisms’ systematic activities; thus, they have been recommended to be much more operative and they show less drug resistance than single targets^4^.

Computational systems biology proposes powerful methodologies to address biomedical queries (e.g., human disease metabolism, the identification of potential drug targets) via a multidisciplinary systems-level study that considers multifaceted interactions between many elements in biological networks^5^. Constraint-based models (CBMs) are very influential in this regard. These models are successfully employed as operative mathematical representations of genome-scale metabolic models (GEMMs) by imposing the governing context- and condition-specific constraints on genome-scale metabolic network reconstructions (GENREs). CBMs can comprehensively analyze metabolic activities and examine the physiological properties of biological systems. Deploying CBMs, systematic analyses can be performed by applying the potent class of computational techniques that are available in constraint-based reconstruction and analysis (COBRA) toolbox^6–8^.

Because in silico studies save significant time and expense, these methods are widely employed to identify the various effects of reaction and gene knockouts on the flux distribution of the metabolic networks of interest. These knockout studies can be implemented to identify new drug targets from three perspectives^9^: (a) targeting virulence factors^3^, (b) metabolite-centric targeting^10–12^, and (c) targeting essential reactions and genes^13–17^. The last perspective is known as the most common method for identifying potential drug targets, and it is not limited to the deletion of only one reaction or gene. Synthetic lethals (SLs) are pairs of non-essential reactions or genes that are deleterious to an organism when they are disrupted simultaneously^18^. Similarly, when the number of targets increases, higher order synthetic lethal sets (n > 2) can be obtained^18^.

We should note that although the identification of higher order synthetic lethal sets can bring in new targets for utilizing different drugs in the design of combinatorial therapeutics, this approach is not common in practice^19^. However, this concept, not certainly by design, might have been deployed already for many drug combination strategies. One example of this strategy is the combination of daptomycin, cefoperazone, and doxycycline for eradication of *Borrelia burgdorferi*, through loss of membrane potential as well as inhibition of energy metabolism, cell wall peptidoglycan synthesis, and protein synthesis^20^. There are other examples in cancer therapeutics such as the combination of BRAF and EGFR inhibitors which effectually influence AKT, MEK and ERK signaling, suggested for colon cancer patients with BRAF mutations^19^. In the mentioned cases, combinatorial therapeutics resulted in more effective impacts compared to monotherapies due to the synergistic effects on different functionalities of the cells.

Two approaches are used to computationally identify SLs: exhaustive search and search space reduction. Exhaustive search is straightforward and has been used in some studies^17,21^, but applying this approach to identify higher order SLs, especially when the cardinality of SLs is greater than three, is not feasible due to computational time problems. Based on the available computational resources, we estimated that the required computational time for the exhaustive search would be over 180 days to obtain all quadruple SLs for *Escherichia coli* using iAF1260^22^ GEMM. Therefore, other methods are required to handle such problems by reducing the search space. Depending on the suggested criteria used to reduce the search space, some of these methods can find only a fraction of the higher order SLs^18^, while some other methods aim to find all the SLs^23–28^.

One of these methods, called “SL Finder,” performs an optimization-based search for the exhaustive and targeted identification of SLs^18^. In order to reduce the search space, this method employs the flux-coupling analysis^29^ to add only one of the fully coupled reactions in the knockout list. This approach was used to discover all double and triple SLs and conduct a targeted identification of a few quadruple and quintuple SLs for iAF1260 GENRE of *E. coli.*

MCSEnumerator finds instead intervention strategies by enumerating the elementary modes of the dual network^30^ of the corresponding metabolic network^23^. It is a powerful approach especially for metabolic engineering applications. Further improvements were made on this approach to obtain the generalized framework of MCSEnumerator and accelerate the dual calculations^24–26^. MCSEnumerator was applied to find all double to quintuple SLs in iAF1260^23^. However, the computational time increases exponentially for SLs that have higher cardinalities, and therefore, the search procedure needs to be stopped after finding a predefined number of SLs or a time limit is reached. Alternatively, in this paper, we propose a targeted enumeration algorithm aiming to increase search efficiency.

Fast-SL is a powerful algorithm that drastically reduces the search space by purging the search space of reactions that are guaranteed not to produce SLs^27^. Fast-SL computes a flux distribution that maximizes the growth rate using a minimum value for the sum of fluxes (${l}_{1}$-norm) in order to identify flux-carrying reactions. In the next step, the algorithm searches only through these flux-carrying reactions, as well as their combinations, to identify SLs within a reduced search space. The authors reported the identification of 127 new synthetic lethal genes in *E. coli*, which had not been found by SL Finder. Also, Fast-SL outperforms the MCSEnumerator by finding the same SLs about four times faster. Fast-SL provided a valuable idea for finding SLs in a reduced search space, but the implementation of this method has two major drawbacks. First, the authors developed different procedures in order to obtain the SLs with different cardinalities, up to quadruple SLs. Therefore, to obtain SLs with more than four targets in each set, an entirely new procedure for each cardinality must be developed. Consequently, if one follows the implementation footsteps in the original Fast-SL, the procedure becomes extremely complicated and requires labor-intensive work to develop. The second drawback is that Fast-SL lacks an organized search method; therefore, several duplicated cases are studied in the original Fast-SL. This causes serious problems when searching for SLs with high cardinalities.

Logic transformation of model (LTM) is another method used in this field. This method changes the stoichiometry matrix (i.e., the S matrix) by adding pseudo-metabolites and reactions to consider the gene-protein-reaction associations (GPRs)^28^. However, the LTM method increases the size of the S matrix, which in turn enlarges the problem size. Thus, more linear programming problems (LPs) must be solved to find SLs. Hence, this method becomes extremely time consuming to perform knockouts regarding higher order SLs.

As mentioned earlier, drug resistance is an important concern and identification of new drug targets based on the concept of synthetic lethality can be a suitable solution for this issue. However, comparing the effects of the different synthetic lethal sets on the metabolic network and its functionalities reveals that some of the sets with higher cardinalities can make stronger and deeper impacts on the network. For instance, we can categorize the synthetic lethal sets into two types: (a) SLs that yield auxotroph strains and (b) SLs that yield strains lacking essential functionalities. The first type of SLs yields strains that are able to restore their growth if the missing nutrients are supplied. In contrast, the strains yielded in the second group cannot restore their growth even if extra components are provided in the growth medium. We expect that the SLs of the second group function more effectively and enable us to aim targets that are harder to resist by pathogens. Based on our in silico observations, higher order SLs provide us with more of these more effective SLs.

The purpose of the current work is to develop a comprehensive and straightforward reimplementation of the Fast-SL algorithm to facilitate the identification of higher order SLs. We call our implementation *Rapid-SL*, which has two major steps that are iteratively performed based on the depth-first search (DFS) algorithm^31^: (1) identification of the seed space (i.e., reactions with nonzero fluxes) and (2) searching within the seed space to find the solutions. The main difference between this new implementation and the original Fast-SL is the compartmentalization of the searching process into several branches. This branching allows embarrassing parallelization^32^ and prevents the examination of duplicate cases. This reduces the search space by about 35–60% compared to Fast-SL. However, in the modern drug discovery process, the target identification is typically the beginning step. Therefore, as in the case of Fast-SL, further analysis on the Rapid-SL results, as a biological hypothesis, is required to reach an approved drug.

In order to examine the performance of the developed method, we compared the results of Rapid-SL and Fast-SL for three microorganisms. Afterwards, we introduced three applications for Rapid-SL that could be effective for the targeted identification of higher order SLs, particularly when the cardinality of SLs is greater than four targets. Accordingly, we can: (1) search among a specific list of reactions chosen consistent with a biological context, (2) apply graph-based search methods, and (3) selectively enumerate the SLs among the DFS branches. Based on our in silico experiments in the current work, over 9000 octuple (*n* = 8) SL reactions were reported for *E. coli* using iAF1260 GEMM. We hope that the identification of higher order synthetic lethal sets using efficient tools such as Rapid-SL paves the way for systematic designing of effective combinatorial therapeutics in future studies.

## Materials and methods

**Figure 1:**
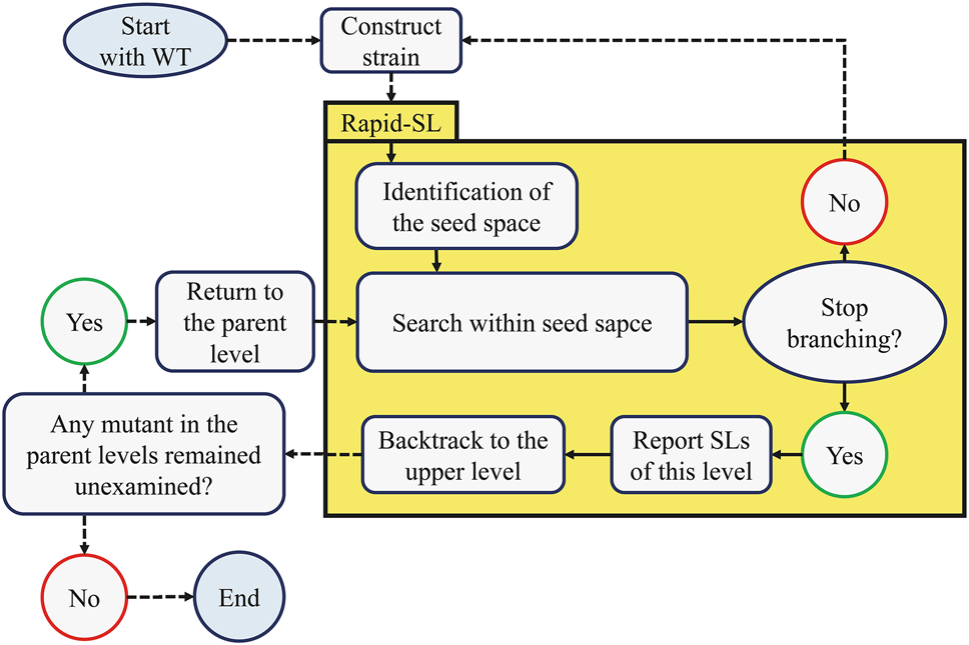
The flowchart of Rapid-SL. Calling Rapid-SL from itself represents the recursive feature of our implementation.

To make the identification of the higher order SLs feasible, we must reduce the search space. The knockout of a reaction set that includes only non-flux-carrying reactions does not change the flux through biomass formation reaction^27^. Therefore, to reduce the search space, we first identify and focus on the set of flux-carrying reactions, which we denote as the *seed space* (J_nz_) in this work. In the second step, we search for the SLs within the seed space. Moreover, each non-lethal subset of the seed space defines a new proliferating mutant strain. Using a DFS approach, we repeat the first and second steps for each of the new mutant strains (Fig. 1). This iterative process continues until certain stopping conditions are met. Each step of the process, as well as the stopping conditions, are described in the following.

### First step: identification of the seed space

We denote the flux of reactions by ν_j_s. In the first step, two flux-balance-analysis (FBA)-related LPs^33^ are considered and solved. These LPs lead to the identification of a flux distribution that maximizes the flux of the biomass objective function (ν_*bio*_), while the ${l}_{1}$-norm of the fluxes is set to its minimum value. The first LP is defined as

1 $$\begin{aligned} & {\text{min}}\mathop \sum \limits_{j} \left| {\nu_{j} } \right| \\ & {\text{s.t}}. \\ & \nu_{bio} = \, \nu_{bio,WT} \\ & {\text{S }}\nu \, = \, 0 \\ & \nu_{{{\text{lb}}}} \le \, \nu \, \le \, \nu_{{{\text{ub}}}} \\ \end{aligned}$$

where ν_*bio,WT*_ is the growth of the wild-type strain calculated by solving the following LP problem:

2 $$\begin{aligned} & {\text{max }}\nu_{bio} \\ & {\text{s}}.{\text{t}}. \\ & {\text{S }}\nu \, = \, 0 \\ & \nu_{{{\text{lb}}}} \le \, \nu \, \le \, \nu_{{{\text{ub}}}} \\ \end{aligned}$$

The goal of computing this flux distribution is to characterize the flux-carrying reactions, or the *seed space*.

Applying flux-variability analysis (FVA)^34^ instead of computing ${l}_{1}$-norm of the fluxes would provide us with more information about the effect of each reaction on the biomass objective function. However, FVA is a time consuming process and using this method repetitively would cripple the whole process.

### Second step: searching within the seed space

**Figure 2:**
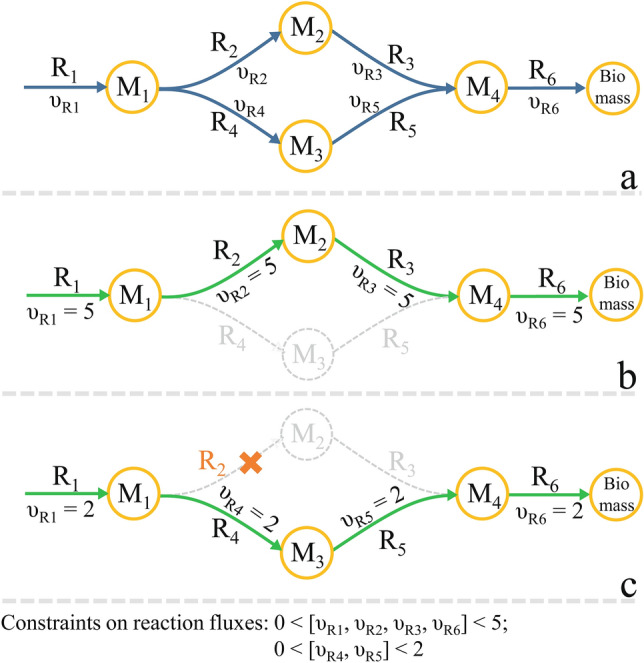
The effect of removing a non-lethal reaction of J_nz_. (**a**) Toy model; R_i_ denotes the reaction names, and υ_Ri_ represents the flux through R_i_. (**b**) Flux distribution of the wild-type strain. J_nz_ = (R_1_, R_2_, R_3_, R_6_). (**c**) Flux distribution of the mutant strain in which R_2_ is removed. In order to maximize the flux of R_6_, the R_4_ and R_5_ reactions gain nonzero fluxes unlike the wild-type strain. Therefore, removing any of the activated reactions R_4_ or R_5_ will block the flux through R_6_ and the biomass objective function.

All combinations of the reactions in the seed space have the potential to form SLs; therefore, the exhaustive search is performed in the second step. However, when an SL is found in this step, the corresponding supersets are excluded to prevent the investigation of duplicated cases or the production of trivial answers. Furthermore, each non-lethal set identified in this step defines a proliferating mutant (i.e., a new virtual strain). This second step also includes the listing of all non-lethal sets to investigate their related proliferating mutants by removing more potential reactions in the next level of the search. Figure 2 depicts these explanations using a toy model. This step is performed in a parallel loop in the first level for the wild-type strain to decrease the wall-clock time.

### Backtracking and the stopping conditions

As described by Pratapa et al.^27^, removing a set of reactions that includes only non-flux-carrying reactions would have no effect on the flux of biomass formation reaction; therefore, at least one reaction in the seed space of the wild-type strain (J_nz_) should participate in each SL. Here, we generalized this statement from the wild-type strain to any virtual strain obtained during our search procedure. In other words, each reaction designated for removal in subsequent steps of the DFS algorithm should originate from the seed space of the parent virtual strain. Therefore, after we evaluate the first and the second steps for the wild-type strain, we iteratively repeat these two steps for all the resulting virtual strains identified in the second step of the previous level. Each of these mutants is treated the same as the wild-type strain; therefore, we face an iterative problem, which is handled using the DFS algorithm (the associated pseudocode is available in Supplementary Note B). Note that, other organized search algorithms such as breadth-first search^35^ and best-first search^36^ instead of DFS can be used easily in our implementation.

The search proceeds from the root node, which consists of a nonlethal set. As an example, consider a general non-lethal set, ∆_m_ with *m* members, which is derived from the evaluation of the second step for the wild-type strain. Let ${\text{J}}_{\text{nz}}^{\Delta{\text{m}}}$ be the seed space of the mutant strain that results from the removal of the non-lethal set of Δ_m_. The set of ${\text{J}}_{\text{nz}}^{\Delta{\text{m}}}$ is evaluated by passing the corresponding mutant to the first step. Because all the reactions in J_nz_ and their combinations are studied in the other branches, only the flux-carrying reactions of this mutant, which belong to J_nz_, are considered at this level. If there are any reactions at this level (i.e., ${\text{J}}_{{{\text{nz}}}}^{{\Delta {\text{m}}}} - {\text{J}}_{{{\text{nz}}}} \ne \emptyset$), the second step is triggered for all the members of ${\text{J}}_{\text{nz}}^{\Delta{\text{m}}} - {\text{J}}_{\text{nz}}$. In Rapid-SL, backtracking occurs in three cases, and extensions cannot go deeper:

- when a set is found to be lethal.
- when no new reaction gains non-zero flux after removing a set (i.e., ${\text{J}}_{{{\text{nz}}}}^{{\Delta {\text{m}}}} - {\text{J}}_{{{\text{nz}}}} = \emptyset$).
- when the size of the examined set reaches the maximum desired cardinality.

Pratapa et al.^27^ state that Fast-SL is not an embarrassingly parallel algorithm; however, they provide a parallel version of the Fast-SL only for the evaluation of quadruple SLs. This parallel version performs parallel calculations for some specific parts of the Fast-SL algorithm. Unlike Fast-SL, Rapid-SL is an embarrassingly parallel algorithm because it is possible to evaluate the branches of the DFS algorithm using a parallel procedure.

**Figure 3:**
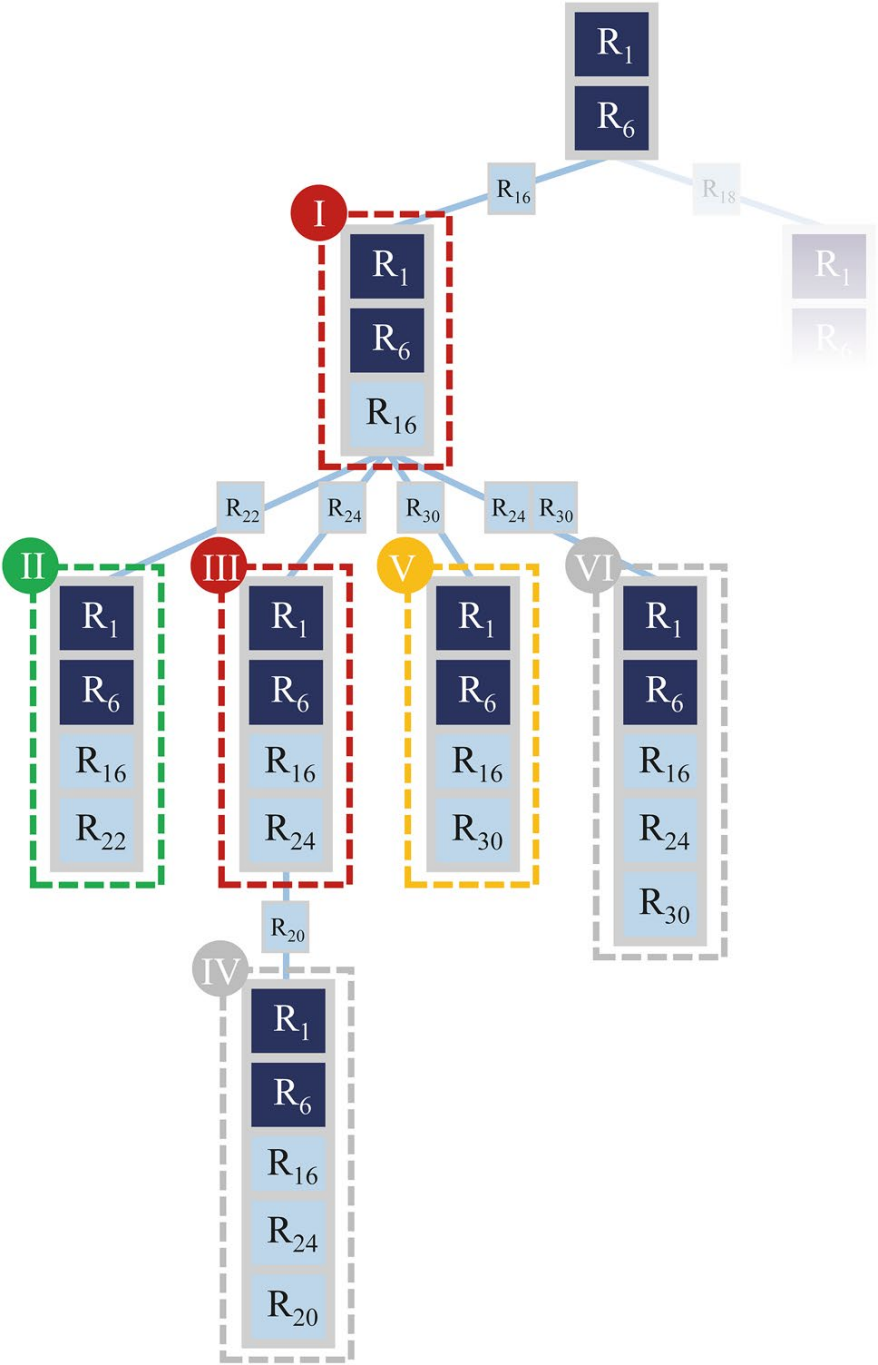
Schematic of a typical example of the depth-first search in our implementation. The squares represent targets. Here (R_1_, R_6_) is a non-lethal set from the second step. The Roman numerals show the order of progress in examining the lethality of different sets. Light blue squares represent the targets that gain non-zero fluxes after removing some reactions. Node I and node III are non-lethal sets and their removal activates new potential targets (). Node II shows a lethal set and thus branching from this node is stopped (). Branching in node V is stopped because no new potential target could be activated (). Branching in nodes IV and VI are stopped because the maximum desired cardinality is reached (). After examining the sets corresponding to the R_16_ branch, the process will continue for the R_18_ branch.

Figure 3 shows an example that illustrates the backtracking process. In this toy example, the maximum cardinality is five (*n* = 5).

### Enumeration of synthetic lethal gene sets

To enumerate the synthetic lethal gene sets, the same procedure is employed, except that those non-zero-flux reactions obtained in each part are converted to the functioning genes using GPR rules (Supplementary Note C). In this work we focused on the enhancement of the identification of SL reactions, which is the main step in the process of finding SL genes. To find SL genes, other improvements can be made by involving and translating GPR rules to make further reduction in the search space prior to the main identification process. Methods such as gMCS^37^ effectively use this feature for identification of synthetic lethal genes.

## Results

We present our results in two parts. First, the performances of Fast-SL and Rapid-SL were compared in the identification of SLs for three microorganisms (see the Supplementary Note D for the comparison between Rapid-SL and duality-based methods). Then, we report the results of the three applications of Rapid-SL for the targeted enumeration of the higher order SLs. The overall computation time of the Fast-SL and Rapid-SL is mostly dependent on the time that is spent on solving the LPs. Therefore, to ensure a fair comparison between Fast-SL and Rapid-SL, we reported the number of LP problems that were solved by each approach. Furthermore, a comparison of wall-clock runtime is provided in Supplementary Note E. The results were obtained using a workstation with a 2.2 GHz Intel Xeon E5-2696 v4 processor, which has 12 cores available for computation.

### Synthetic-lethals of the three microorganisms

Table 1 shows the respective numbers of SLs with different cardinalities (up to quadruples) obtained by our implementation and obtained by the original Fast-SL. Since the SLs identified by the both methods were found to be the same, the table does not report the number of these SLs found by each method.

**Table 1:** Comparison of the number of LPs solved by Rapid-SL vs. Fast-SL for three GEMMs.

	*Escherichia coli*	*Salmonella Typhimurium*	*Mycobacterium tuberculosis*
Model name	iAF1260^22^	STM_v1.0^14^	iNJ661^38^
Medium	iM9/glucose	iM9/glucose	Middlebrook 7H9
Number of reactions	2382	2546	1028
Number of exchange and diffusion reactions^a^	331	378	86
Number of reactions in J_nz_	406	484	414
Single lethal reactions	278	329	309
Lethal reaction pairs	96	152	75
Lethal reaction triplets	247	275	140
Lethal reaction quadruplets	402	1008	463
**Total number of LPs solved**			
Fast-SL	1.45 ‌‌‌$\times$ 10^7^	3.01 $\times$ 10^7^	1.19 $\times$ 10^7^
Rapid-SL	7.35 $\times$ 10^6^	1.98 $\times$ 10^7^	4.90 $\times$ 10^6^

^a^The exchange and diffusion reactions are not generally considered in the lethality analysis.

Table 1 indicates that our new implementation explores about 40–65% of the search space of the original Fast-SL, and it does not omit any potential cases (Supplementary Files S1–S3). This reduction in the search space is achieved by preventing the investigation of identical cases produced in different branches.

### Applications of Rapid-SL

As the maximum desired cardinality of SLs increases, there is an exponential increase in both the search space and the number of cases to be examined in order to find all possible SLs. As a result, it is not feasible to find all possible SLs with high cardinalities (e.g., octuple SLs) using the algorithms that are currently available^23,27^. Therefore, we take advantage of our new implementation to effectively investigate these large search spaces. Here we introduce three applications of Rapid-SL to perform the targeted enumeration of higher order SLs.

#### Searching a list of specific targets

The simplest method to find a fraction of solutions is to specify only a limited group of reactions. However, it is not clear what reactions should be selected. These reactions may be selected from a specific subsystem or pathway that has been diagnosed as important for the growth of the microorganism. For example, we performed a search to find octuple SLs (i.e., with eight reactions in a set) among 65 core reactions introduced by Hädicke and Klamt for generating a core model from iJO1366^39,40^. The results obtained in this application are presented in Supplementary File S4.

Our new implementation makes this analysis feasible, but at the first sight, it may seem that using Rapid-SL is not necessary, and it may seem sufficient to find these results using an exhaustive search because of the small number of reactions involved in the analysis. However, Rapid-SL uses a search space that is about 50 times smaller than the exhaustive search, which consequently requires an extremely time-consuming process even for small numbers of reactions. Also, it is not feasible to perform this analysis using the original Fast-SL, because separated algorithms should be devised for the cardinality of each SL.

#### Applying constraints on the branching of the DFS

Since Rapid-SL applies the DFS algorithm to investigate the search space, it is possible to define thresholds or conditions to limit the branching and search only the more probable parts of the corresponding tree. For example, we established a criterion in which sets are allowed to branch only if their deletion reduces the growth rate of the corresponding strain by at least 1%. The results obtained by applying this criterion to the process of identifying octuple SLs (*n* = 8) for iAF1260 are presented in the Supplementary File S5. Here, the critical value of 1% was selected based on trial and error. Other values could be employed based on the studied GEMM and the growth medium. Also, other types of constraints could be defined, such as the change in the pool of a specific metabolite or the fluxes of other reactions.

#### Selective enumeration among the DFS branches

Consider the process of seeking the quadruple SLs of *E. coli* using iAF1260. If we group the branches of the Rapid-SL algorithm based on the number of reactions in the starting node of each branch, it is evident that the number of LPs solved in each group substantially increases as the cardinality of the starting node increases. On the other hand, the number of identified SLs per LP solved dramatically decreases (Table 2). Therefore, a large portion of the SLs can be identified by performing a lethality analysis on a limited number of branches.

**Table 2:** The number of SLs and corresponding LPs solved in each group of branches of the Rapid-SL, while searching for single lethals to quadruple SLs in iAF1260. The branches are grouped based on the number of members in their starting node. Evaluating only the first group of branches identifies over 34% of SLs, while only about 0.65% of LPs must be examined.

Group identifier	Cardinality of the starting node	Number of SLs identified	Number of LPs solved
I	One reaction	254	47,869
II	Two reactions	220	646,501
III	Three reactions	192	2,679,417
IV	Four reactions	79	3,974,889

According to Table 2, it is possible to extend only the branches in Group (I) to identify over 34% of all SLs (excluding single lethals), while about 0.65% of all LPs are examined. It should be noted that 254 SLs identified in Group (I), consist of 74 double, 98 triple, and 82 quadruple SLs. The same analyses were performed for the same microorganism (*E. coli*) with a different genome-scale model (i.e. iJO1366) and also for a different type of microorganism (i.e. *Klebsiella pneumoniae*, iYL1228^41^) to check the generalizability of this observation (Table 3).

**Table 3:** SLs identified by evaluation of only the branches with one reaction in the starting node (Group I).

Model	Cardinality of SLs	All SLs	Enumerated in Group (I)	Fraction (%)
iJO1366	Double SL	259	237	91.5
	Triple SL	1162	871	75.0
	Quadruple SL	3585	2275	63.5
	Total SLs	5006	3383	67.6
	Total number of LPs solved	32,615,092	322,670	1.0
iYL1228	Double SL	146	127	87.0
	Triple SL	289	178	61.6
	Quadruple SL	1090	513	47.1
	Total SLs	1525	818	53.6
	Total number of LPs solved	12,283,617	125,159	1.0

It could be inferred from Table 3 that an evaluation of the branches in Group (I) is a reliable approach to find a considerable fraction of all SLs. For the GEMMs that were studied, we found up to 67% of all SLs (i.e. including double, triple and quadruple SLs) using the illustrated method by examining only about 1% of the search space that must be evaluated to find all quadruple SLs. We applied this method to find the octuple SLs of iAF1260 to investigate the efficiency of this approach for identification of higher order SLs with more than four targets in each set (Supplementary File S6).

Table 4 summarized the results of the three applications of Rapid-SL and according to this table, over 9000 octuple SLs were found using the illustrated application of Rapid-SL. Based on the size of the GEMM and the maximum desired cardinality of SLs, it is possible to consider other groups of branches. For instance, Table 2 shows that evaluating both Groups (I) and (II) for iAF1260 reduces the search space by over 90% while identifying over 63% of all SLs.

**Table 4:** Results of three introduced applications.

	Search through a specific list of targets	Applying constraints on the branching of the DFS	Selective enumeration among the DFS branches
Model name	iJO1366	iAF1260	iAF1260
Medium	iM9/glucose	iM9/glucose	iM9/glucose
Double SLs	11	10	74
Triple SLs	18	27	98
Quadruple SLs	68	41	82
Quintuple SLs	22	11	159
Sextuple SLs	86	59	307
Septuple SLs	319	125	476
Octuple SLs	681	402	9126
Total number of LPs solved	78,297,112	27,803,258	89,958,961

## Discussion

In this paper, we introduce Rapid-SL as a new implementation of Fast-SL that enables the algorithm to find higher order SLs with arbitrary cardinalities. Unlike Fast-SL, this new implementation fully supports *embarrassingly* parallel computations. Furthermore, compared to Fast-SL, the application of the DFS algorithm (a structured search method) decreased the number of evaluated LP problems by about 35–60%. The original implementation of Fast-SL is not embarrassingly parallel and suffers from time consuming sequential computations in some of its steps. Accordingly, for larger models and higher order SLs, the difference between the computational time of Fast-SL and Rapid-SL increases and Rapid-SL becomes more and more efficient. Although Rapid-SL is not limited in terms of the cardinality of SLs, it is not feasible to seek for all SLs with higher cardinalities, especially when n > 4, without using computer clusters. When using a single conventional computer, the runtime of this examination may extend to several months because of the tremendous number of potential cases. Owing to our proper implementation, Rapid-SL can effectively find a considerable portion of higher order SLs by searching only a relatively small fraction of potential cases. Accordingly, three Rapid-SL applications were introduced: (a) searching among a selected list of potential reactions, (b) applying constraints on the branching of the DFS, and (c) selective enumeration among the DFS branches. These applications identified up to 67% of quadruple SLs by searching about 1% of the potential cases. Particularly, over 9,000 octuple synthetic reactions were found for iAF1260 in the third application. Accordingly, Rapid-SL can be effective for investigating large models such as genome-scale metabolic models of human cells to find drug targets with high cardinalities.

Although the first two applications find fewer SLs than the other application, they may still be useful for seeking SLs with specific biological considerations. The importance of this feature becomes clearer noting that a single organism may have more than several thousands SLs with high cardinalities, and experimental validation of all of these SLs is not feasible due to the immense number of required experiments. Therefore, the both scenarios require the consideration of biological criteria when searching for useful SL sets. In future work, we will focus on defining new criteria to reduce the number of potential drug-targetable SLs.

## Conclusion

Although the combinatorial therapeutics are expected to be effective against drug resistance pathogens and higher order SLs can potentially nominate candidates for simultaneously attacking multiple targets, it is still challenging to determine which combination would be practical and most useful. For example, possible negative synergistic effects narrow down the practical drug combinations. Furthermore, when the number of targets in an SL increases, the chance of finding a set with all druggable targets decreases. Therefore, it would be desirable to devise a systematic pipeline to investigate the identified higher order SLs as the starting point and derive a combinatorial therapeutic design as the outcome.

## Supplementary Information

Supplementary Information 1.

Supplementary Information 2.

Supplementary Information 3.

Supplementary Information 4.

Supplementary Information 5.

Supplementary Information 6.

Supplementary Information 7.

Supplementary Information 8.

## Data Availability

Rapid-SL is publicly available at https://github.com/CSBLaboratory/RapidSL (Supplementary File S7).
