# Supplementary figures and images for: Rapid-SL identifies synthetic lethal sets with an arbitrary cardinality
Source: Sci Rep. 2022 Aug 18;12:14022. doi: 10.1038/s41598-022-18177-w (PMC9388495; doi:10.1038/s41598-022-18177-w)

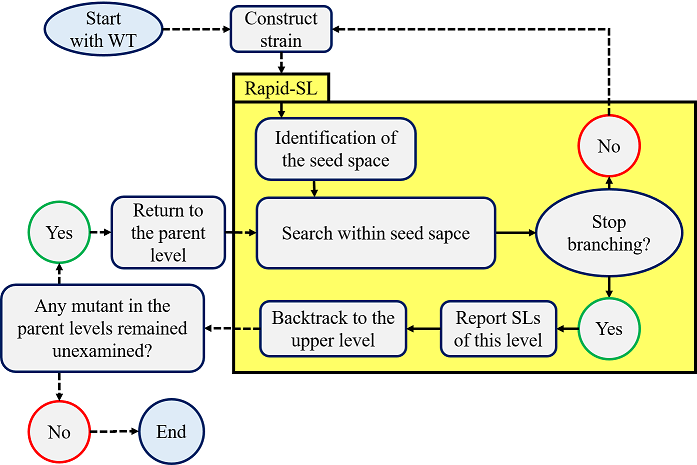

Supplement: Supplementary file 7 — Supplementary Information 7. [file 41598_2022_18177_MOESM7_ESM.zip › RapidSL-main/RapidSL_abstract_flowchart.png]
