# Supplementary material for: Rapid-SL identifies synthetic lethal sets with an arbitrary cardinality
Source: Sci Rep. 2022 Aug 18;12:14022. doi: 10.1038/s41598-022-18177-w (PMC9388495; doi:10.1038/s41598-022-18177-w)
Supplement: Supplementary file 8 — Supplementary Information 8. [file 41598_2022_18177_MOESM8_ESM.docx]

**Rapid-SL identifies synthetic lethal sets with an arbitrary cardinality**

Mehdi Dehghan Manshadi, Payam Setoodeh, Habil Zare

**Supplementary Notes**

**A. Supplementary files description:**

**Supplementary_file_S1:** The results of the lethality analysis for *Escherichia coli*, iAF1260 model, for the maximum cardinality of n = 4.

**Supplementary_file_S2:** The results of the lethality analysis for *Salmonella Typhimurium*, STM_v1.0 model, for the maximum cardinality of n = 4.

**Supplementary_file_S3:** The results of the lethality analysis for *Mycobacterium tuberculosis*, iNJ661 model, for the maximum cardinality of n = 4.

**Supplementary_file_S4:** The lethal sets of *E. coli*, iJO1366 model, found in the first application of Rapid-SL. The analysis was performed for the maximum cardinality of n = 8.

**Supplementary_file_S5:** The lethal sets of *E. coli*, iAF1260 model, found in the second application of Rapid-SL. The analysis was performed for the maximum cardinality of n = 8.

**Supplementary_file_S6:** The lethal sets of *E. coli*, iAF1260 model, found in the third application of Rapid-SL. The analysis was performed for the maximum cardinality of n = 8.

**Supplementary_file_S7:** MATLAB codes for Rapid-SL.

**B. Pseudocode of Rapid-SL:**

a) *SearchWithinSeedspace* is the second step of Rapid-SL which finds synthetic lethals for the list of *Seed Space.* The list of *SeedSpace* is defined in the first step of Rapid-SL and embedded in the RapidSL function.

1: **function** SearchWithinSeedspace (*model*, *maxCardinality, SeedSpace*, *Cutoff, grRate*)

2: **Input**: *model*: metabolic model,

*maxCardinality*: maximum cardinality for this level

*SeedSpace:* seed space of the *model*

*Cutoff:* the cut off threshold for lethality

*grRate:* the growth rate corresponding to the *model*

3: **Output:** lethal sets, non-lethal sets and the growth rate corresponding to non-lethal sets of the *model*

4: Identify *Candidates* ## The list of single reactions that should be removed from the mutant

5: Initialize *lethals*  and *nonLethals*  ## Each is an empty list

5: **for** i = 1 : maxCardinality

6: **for** j = 1 : the number of cases in *Candidates*

7: Compute *mutantStrain* ## The upper and lower bounds of all reactions in set *Candidates(i)* are set to zero

6: *grRate* = FBA (*mutantStrain*) ## It returns the maximum growth rate related to the mutant strain

7: **if** *grRate* < *Cutoff*

8: Add *Candidates(i)* to *lethals*

9: **else**

10: Add *Candidates(i)* to *non-lethals*

11: Update *Candidates* ## Update the list of reaction sets that should be removed in the next iteration

12: Identify *trivialSolution* ## The list of Candidates that are supersets of the identified lethal sets

13: *Candidates = Candidates – (Candidates ∩ trivialSolution)*

14: **end** of for

16: **end** of for

17: Return *lethals*, *nonLethals*

b) *RapidSL* employs the first and the second steps using the depth first search algorithm to find all synthetic lethals.

1: **function** RapidSL (*model*, *maxCardinality,* *Cutoff, eliList*)

2: **Input**: *model*: metabolic model,

*maxCardinality*: maximum cardinality for this level.

*Cutoff:* the cut off threshold for lethality.

*eliList:* the set of all reactions that should be excluded from the lethality analysis.

3: **Output:** Synthetic lethal sets of the strain

## The next four lines define the **first step** and calculate the *SeedSpace* and *grRate*

4: *grRate* = FBA (*model*) ## It returns the maximum growth rate related to the mutant strain.

5: *Flux* = FBA (*model*, *grRate)* ## It returns the taxicab solution corresponding to *grRate.*

6: Identify *Jnz* ## The list of reactions with non-zero fluxes in the strain.

7: *SeedSpace* = *Jnz ∩* *eliList*

8: Run SearchWithinSeedspace (*model*, *maxCardinality, SeedSpace*, *Cutoff, grRate*) to get *Lethals* and *non-Lethals*

9: **if** stopping conditions are met

10: define *SLs* as an empty variable

11: **else**

12: Identify *mutant*S*train*s ## the upper and lower bounds of all reactions in the *set* are changed to zero

13: **for** *set* in *nonLethals*:

14: Run RapidSL (*model*, *maxCardinality,* *Cutoff, eliList*) to obtain *SLs* ## Branching of the Depth first search algorithm

15: **end** of for

16: *SLs* = *SLs* ∪ *Lethals*

17: Return *SLs*

**C. Comments on gene lethality analysis using Rapid-SL:**

Here we compare the gene based Rapid-SL and Fast-SL:

| Rapid-SL | Fast-SL |
| --- | --- |
| Pros:   1. No limit for cardinality 2. Promising for finding all SLs 3. Graph based searches can be used for effective seeking for SLs,   Cons:   1. Solves more LPs compared to Fast-SL | Pros:   1. Solves less LPs compared to Rapid-SL   Cons:   1. There is no function for finding quintuple or higher order synthetic lethal genes. 2. It is possible to miss some cases due to its implementation. |
|  |  |
|  |  |

Gene-based Fast-SL finds synthetic lethal genes by searching and finding synthetic lethal reactions. In other words, Fast-SL finds synthetic lethal reactions and then converts the reaction sets to their associated gene sets. Although this method reduces the search space, it is possible to miss lethal gene sets that target a larger number of independent reactions. On the other hand, Rapid-SL directly relates the flux carrying reactions to the corresponding genes and then analyzes the effects of their removal. This method forces the examination of more cases but it promises that no synthetic lethal gene set will be missed. For example, if one uses Fast-SL to obtain all double synthetic lethal genes for *Klebsiella pneumoniae*, iYL1228, the case of {KPN_00456, KPN_02238} remains undiscovered. It happens because this set codes 6 reactions and Fast-SL does not consider this set of reactions in its analysis. Note that this kind of lethal gene set may be assumed a valuable potential drug target because by targeting only a few genes, a large number of reactions will be crippled.

To sum up, in the case of gene-based lethality analysis, users can deploy Fast-SL instead of Rapid-SL if the maximum desired cardinality is less than 5 and only if **missing** some SL sets is not important; otherwise, we strongly recommend using Rapid-SL for gene-based lethality analyses.

**D. Comparison of computational time between Rapid-SL and duality-based methods using iML1515:**

Pratapa et al. [1] showed that Fast-SL is about 4 times faster than MCSEnumerator. Since Rapid-SL outperformed Fast-SL, here we only compare the respective performances of Rapid-SL and CNAMCSEnumerator2 [2] for the identification of triple SLs of iML1515 [3].

| Cardinality | Rapid-SL | CNAMCSEnumerator2 |
| --- | --- | --- |
| Single Lethal | 271 | 271 |
| Double Lethal | 304 | 304 |
| Triple Lethal | 1287 | 1287 |
| Time | 19 minutes | Over 20 hours |

Note that, although the process of finding quadruple lethal sets did not finish after 30 days using MCSEnumarator2, 4056 quadruple lethals were obtained after 143 hours (about 6 days) using Rapid-SL.

**E. Comparison of computational time between Fast-SL and Rapid-SL using iJO1366:**

Because Fast-SL uses sequential computations in some steps and it is not embarrassingly parallel [1], for larger models and higher SL sets the difference between runtimes of Fast-SL and Rapid-SL increases and Rapid-SL becomes more and more efficient. For example, for obtaining the quadruple SLs for *E. coli* using iJO1366, the overall computational time is 99 and 35 hours for Fast-SL and Rapid-SL, respectively. This implies 65% reduction in computational time.

**References**

[1] A. Pratapa, S. Balachandran, and K. Raman, "Fast-SL: an efficient algorithm to identify synthetic lethal sets in metabolic networks," *Bioinformatics,* vol. 31, no. 20, pp. 3299-3305, 2015.

[2] P. Schneider, A. von Kamp, and S. Klamt, "An extended and generalized framework for the calculation of metabolic intervention strategies based on minimal cut sets," PLoS computational biology, vol. 16, no. 7, p. e1008110, 2020.

[3] J. M. Monk et al., "iML1515, a knowledgebase that computes Escherichia coli traits," Nature biotechnology, vol. 35, no. 10, pp. 904-908, 2017.
